# Perioperative systemic steroid for rapid recovery in total knee and hip arthroplasty: a systematic review and meta-analysis of randomized trials

**DOI:** 10.1186/s13018-017-0601-4

**Published:** 2017-06-27

**Authors:** Chen Yue, Rong Wei, Youwen Liu

**Affiliations:** 1 Department of Orthopedic Surgery, Luoyang Orthopedic Hospital of Henan Province. Orthopedic Hospital of Henan Province, 82# QiMing Road, 471000 Luoyang, Henan Province China; 2 Department of Orthopedic Surgery, Luoyang Central Hospital Affiliated to Zhengzhou University, 471000 Luoyang, Henan Province China

**Keywords:** Systemic steroid, Rapid recovery, Knee and hip arthroplasty

## Abstract

**Background:**

Perioperative systemic steroid administration for rapid recovery in total knee and hip arthroplasty (TKA/THA) is an important and controversial topic. We conducted this systematic review and meta-analysis to evaluate the overall benefits and harms of perioperative systemic steroid in patients undergoing TKA and THA.

**Methods:**

A comprehensive search was performed on PubMed, OVID, and Web of Science databases, and a systematic approach was carried out starting from the PRISMA recommendations. Relevant randomized controlled trials (RCTs) were selected. The risk of bias was evaluated according to the Cochrane Handbook for Systematic Reviews of Interventions version. Data were extracted and meta-analyzed or qualitatively synthesized for all the outcomes.

**Results:**

Data were extracted from 11 trials involving 774 procedures. Meta-analysis showed that high-dose systemic steroid (dexamethasone > 0.1 mg/kg) rather than low dose is effective to reduce postoperative nausea and vomiting and postoperative acute pain (within 24 h). In addition, systemic steroid is associated within faster functional rehabilitation and greater inflammation control. On the other hand, systemic steroid is associated with a higher level of postoperative serum glucose on the operation day. The complications between groups are similarly low.

**Conclusions:**

Our study suggests that by providing lower incidence of postoperative nausea and vomiting and less postoperative acute pain, high-dose systemic steroid plays a critical role in rapid recovery to TKA and THA. The preliminary results also show the superior possibility of systemic steroid in functional rehabilitation and inflammation control. More large, high-quality studies that investigate the safety and dose–response relationship are necessary.

**Electronic supplementary material:**

The online version of this article (doi:10.1186/s13018-017-0601-4) contains supplementary material, which is available to authorized users.

## Background

For patients with diseased knee or kip joints, total knee or hip arthroplasty (TKA/THA) is considered as one of the most effective procedures for relieving pain and improving joint function [1, 2]. Improvements in joint arthroplasty procedures will routinely witness rapid recovery, faster functional recovery, lower postoperative discomfort, and higher patient satisfaction, which is obviously a win-win for patients and surgeons [3, 4].

Pain and nausea are considered as serious impediments to rapid recovery in joint arthroplasty [4]. Poor pain relief, as well as postoperative nausea and vomiting (PONV), can lead to strong discomforts, emotional distress and low satisfactions [5, 6]. An effective control of pain and PONV will play a critical role in rapid recovery to TKA and THA.

Steroid, or glucocorticoid, is a kind of anti-inflammatory drug which provides effective pain relief by reducing inflammation at the site of surgical trauma [7]. In addition, it reduces PONV by exerting a central antiemetic effect by inhibiting prostaglandin synthesis and the release of endogenous opioids [4, 8, 9]. Perioperative systemic steroid has been used to prevent PONV and ease the pain in general surgery, and the excellent analgesic and antiemetic benefits have clearly been approved [10, 11]. In recent years, it has been gradually applied in TKA and THA, but the effectiveness and safety of systemic steroid is still controversial, especially concerns for the potential side effects. On one hand, some studies suggested perioperative systemic steroid to be associated with lower perceived perioperative pain and less risk of PONV [12, 13], and some studies showed superior outcomes in function rehabilitation and even in postoperative thrombogenic markers levels with systemic steroid [14, 15]. On the other hand, some studies have found that there are no significant benefits of steroid in improving function and reducing the incidence of PONV [16, 17]. In addition, some studies also reported potential adverse events with steroid, such as infection, poor sleep quality, and increased serum glucose level in early postoperative phase [14].

The highest level-evidence assessment of clinical interventions should be evidence-based, relying on results from update meta-analyses of randomized controlled trials. But, as of this time, we are not aware of any published meta-analyses evaluating the overall benefits and harms of systemic steroid in TKA and THA. Therefore, we carried out this meta-analysis to give an evaluation of the update clinical evidence. Specifically, we systematically reviewed randomized controlled trials (RCTs) assessing the possible benefits or harms of perioperative systemic steroid administration for TKA and THA, and we evaluated available data on seven categories of outcomes: pain, PONV, complications and adverse events, functional outcome, hospital length of stay (LOS), inflammatory factors, and thrombogenic markers.

## Methods

This was a review of existing literature and did not involve any handling of individual patient data, so the ethical approval was deemed unnecessary.

### Search methodology

Two reviewers (CY and RW) independently searched PubMed, OVID, and Web of Science databases in February 2017 without restrictions on publication date or language. Search terms included “total hip arthroplasty”, “total hip replacement”, “total knee arthroplasty”, “total knee replacement”, “THA”, “THR”, “TKA”, “TKR”, “steroid”, “glucocorticoid”, “dexamethasone”, “Corticosteroid”, “Cortisone”, “Methylprednisono”, “Prednisone”, and “Betamethasone”. Search terms were combined using the Boolean operators ‘AND’ or ‘OR’. Reference lists of relevant articles were manually searched to identify additional trials.

### Selection criteria

A study was considered eligible for inclusion if it (1) was an RCT, (2) was based on the intervention with intravenous or intramuscular or oral steroid, and (3) reported data for at least one of the following outcomes: pain, PONV, functional outcome, hospital length of stay, inflammatory factor, thrombogenic markers, and complications. Studies were excluded if steroid was used as intra-articular injection or nerve block, or applied in hemiarthroplasty, unicompartment knee arthroplasty, arthroscopic surgery, or revision THA/TKA.

### Literature selection

All potential studies were imported into Endnote X7 and duplicates were excluded. Then, two researchers (CY and RW) independently excluded studies based on titles and abstracts. At last, by reading the full text carefully, the two researchers eliminated the studies that did not satisfy the selection criteria. Disagreements were resolved by discussion with a third researcher (YWL).

### Data extraction and assessment of study quality

The same two researchers independently extracted the following items: first author’s name, publication year, country, sample sizes, follow-up period, and anesthesia types. Data were collected on the following primary outcomes: pain, PONV, and complications, and adverse events. Data were also collected on the following secondary outcomes: functional outcome, hospital length of stay, inflammatory factors, and thrombogenic markers.

Working independently, two researchers (CY and RW) assessed the risk of bias according to the Cochrane Handbook for Systematic Reviews of Interventions version [18], which includes the following seven items: sequence generation, allocation concealment, blinding of participants, blinding of outcome assessor, incomplete outcome data, reporting bias, and other bias. Discrepancies between assessments were resolved by discussion with a third reviewer when necessary.

### Statistical analysis

Outcomes for which data could not be compared directly across studies were synthesized qualitatively, as were outcomes for which insufficient data were reported across studies. Outcomes for which sufficient, equivalent data were reported across studies were meta-analyzed using Forest plots generated with RevMan 5.2. Dichotomous data were expressed as proportions, such as PONV and complications; the intervention effect was expressed as the Odds Ratio. Continuous data reported in the same way across studies, such as for hospitalization, were meta-analyzed in terms of the weighted mean difference (WMD) and associated 95% confidence interval (95% CI). Continuous data reported in different ways across studies were meta-analyzed in terms of the standardized mean difference (SMD). Continuous data reported as medians and ranges were transformed into means and standard deviations using Hozo’s formula [19].

Pooled data were assessed for heterogeneity using the chi-squared test and I-squared tests. Heterogeneity was defined as absent when I^2^ was between 0 and 25%; low, between 25.1 and 50%; moderate, between 50.1 and 75%; or high, between 75.1 and 100%. Fixed-effects meta-analysis was performed when p ≥ 0.1 and I^2^ ≤ 50%; otherwise, random-effects meta-analysis was performed.

## Results

### Search results

**Fig. 1:**
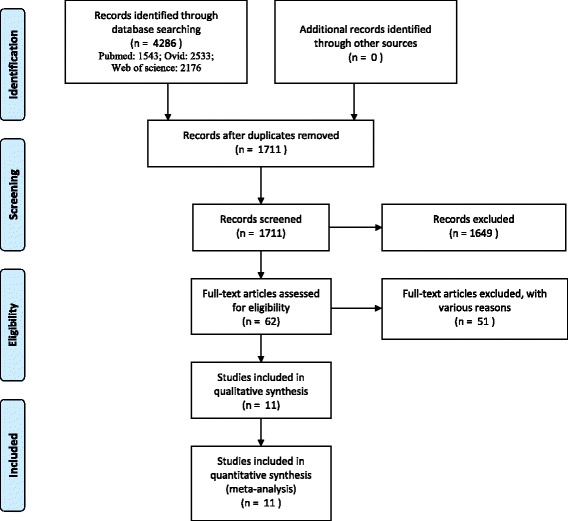
Flowchart of study selection

A total of 6252 studies were identified and imported into Endnote X7 after a systemic search of Pubmed, OVID and Web of Science (Additional file 1: Figures S1-S3 illustrates the detailed search histories for each electronic database), and no additional records were found during manual searches of references. 1711 studies were left after removal of duplicates, and then another 1649 studies were excluded based on their titles and abstracts. The remaining 62 studies were assessed by reading the full texts, and 51 were removed for failing to satisfy the selection criteria. Finally, 11 studies were considered appropriate and were included in this systematic review and meta-analysis. The details of study identification, screening, and selection are listed in Fig. 1.

### Description of included studies

The included 11 studies involved 774 cases of participants [12–17, 20–24]. Five studies were performed in the USA, two in Denmark, two in Canada, and one each in Japan and Korea. Nine studies provided detailed follow-up period, ranging from 1 month to 1 year [12, 14, 16, 17, 20–24]. Eight studies evaluated the incidence of PONV [12–14, 16, 20–23]. Nine studies evaluated postoperative pain based on VAS or NRS scale [12, 14, 16, 17, 20–24]. All the studies reported perioperative surgical complications and four studies reported the potential perioperative adverse events [14–16, 22]. Four studies evaluated postoperative function used patient-reported functional scales or range of motion [14, 16, 17, 21]. Six studies provided data on hospitalization time [14, 16, 20–23]. Six studies analyzed the levels of inflammatory factors of CRP or IL-6 [14, 16, 20, 22–24]. One study each assessed the levels of thrombogenic markers in TKA [15] or THA [22]. Tables 1 and 2 show the detailed descriptions, and Table 3 shows the risks of bias assessment of included studies.

**Table 1:** Detailed descriptions of included studies

Author (Year)	Surgery	Size (E/C)	Delivery way	Dose of steroid	Anesthesia	Country	Follow-up
Koh et al. (2013)[12]	TKA	269 (135/134)	IV	10 mg DA before operation and further dose at end of operation (H)	SA	Korea	1 year
Fujii et al. (2005) [13]	TKA	80 (20/20/20/20)	IV	4 mg (L) or 8 mg (H) or 16 mg (H) DA at end of operation	GA or SA	Japan	Unclear
Jules-Elysee et al. (2011) [14]	TKA	30 (15/15)	IV	100 mg HC before operation and futher dose at 8 h later (L)	SA	USA	6 months
McLawhorn et al. (2014) [15]	TKA	23 (11/12)	IV	100 mg HC before operation and followed by 2 doses each 8 h apart (H)	SA	USA	Unclear
Jules-Elysee et al. (2012) [16]	TKA	34 (17/17)	IV	100 mg HC before operation and further dose at 8 h later (L)	SA	USA	6 months
Bergeron et al. (2009) [17]	THA	50 (25/25)	IV	40 mg DA before operation (H)	SA	Canada	1 year
Lunn et al. (2011) [20]	TKA	48 (24/24)	IV	125 mg MP before operation (H)	SA	Denmark	1 month
Backes et al. (2013) [21]	TKA/ THA	TKA:68 (28/25/20) THA:47 (13/17/17)	IV	E1: 10 mg DA before operation (H) E2: 10 mg DA before operation and further dose at 24 h later (H)	GA	USA	6 months at least
Sculco et al. (2015) [22]	THA	27 (13/14)	PO + IV	20 mg PD orally before operation, followed by 2 doses 100 mg HC intravenously each 8 h apart (H)	SA	USA	3 months
Lunn et al. (2013) [23]	THA	48 (24/24)	IV	125 mg MP before operation (H)	SA	Denmark	1 month
Kardash et al. (2007) [24]	THA	50 (25/25)	IV	40 mg DA before operation (H)	SA	Canada	1 month

*E* experimental group, *C* control group, *IV* intravenous injection, *PO* oral administration, *DA* dexamethasone, *HC* hydrocortisone, *PD* prednisone, *MP* methylprednisolone, *SA* spinal anesthesia, *GA* general anesthesia, *H* high-dose steroid, dexamethasone > 0.1 mg/kg or other equivalent steroid, *L* low-dose steroid, dexamethasone ≤ 0.1 mg/kg or other equivalent steroid

**Table 2:** Summary of significantly different outcomes between groups

First author (Year)	Surgery	Outcome assessments	Summary of significantly different outcomes between groups
Koh et al. (2013) [12]	TKA	C, P, PONV	Less PONV and pain in DA group, and no significant difference in complications
Fujii et al. (2005) [13]	TKA	C, PONV	8 mg and 16 mg DA causes Less PONV, but 4 mg DA doesn’t work
Jules-Elysee et al. (2011) [14]	TKA	C, F, I, L, P, PONV	Lower IL-6 level within 12 hrs after surgery, better ROM at discharge, but a higher serum glucose level on arrival to the PACU in HC group, no difference in pain relief, PONV, LOS and complications
McLawhorn et al. (2014) [15]	TKA	C, L, T	Lower levels of PAP and PF1.2 while a higher serum glucose level within the first day after surgery in HC group, no significant difference in complications and LOS
Jules-Elysee et al. (2012) [16]	TKA	C, F, I, L, P, PONV	Less pain and lower IL-6 level within 24 hrs after surgery, better ROM at discharge, but a higher serum glucose level within the first day after surgery in HC group, no difference in PONV, LOS and complications
Bergeron et al. (2009) [17]	THA	C, F, P	Similar results in pain, HHS scores and complications between groups at 6 weeks and 1 year postoperatively
Lunn et al. (2011) [20]	TKA	C, I, L, P, PONV	MP causes better outcomes in pain, CRP levels and PONV events, but no difference in complications and LOS
Backes et al. (2013) [21]	TKA/ THA	C, F, L, P, PONV	Significant pain relief, faster functional recovery, less PONV and earlier discharge in DA groups
Sculco et al. (2015) [22]	THA	C, I, L, P, T,	Less severe pain and lower IL-6 level while a higher serum glucose level in steroid group, no difference in the levels of PAP and PF1.2, LOS and complications
Lunn et al. (2013) [23]	THA	C, I, L, P, PONV	MP causes better outcomes in pain, CRP levels and PONV events, but no difference in complications and LOS
Kardash et al. (2007) [24]	THA	C,I,P, PONV	Less pain, PONV and lower CRP level in DA group, no difference in complications

C: Complications and adverse events; F: Functions; I: Inflammatory factor

L: LOS, Length of stay; T: Thrombogenic markers; P: Pain

PONV: Postoperative nausea and vomiting

DA: dexamethasone; HC: Hydrocortisone; PD:prednisone; MP methylprednisolone

PAP: plasmin-alpha-2-antiplasmin complex, a kind of thrombogenic markers

PF1.2prothrombin fragment1.2, a kind of thrombogenic markers

CRP: C-reactive protein; IL-6: Interleukin-6

HHS: Harris hip score; ROM: Range of motion

**Table 3:** Risk of bias of included studies

Author (year)	1	2	3	4	5	6	7
Koh et al. (2013) [12]	+	+	+	+	+	?	?
Fujii et al. (2005) [13]	+	+	+	?	?	?	?
Jules-Elysee et al. (2011) [14]	+	+	+	+	?	+	?
McLawhorn et al. (2014) [15]	+	+	+	?	+	?	?
Jules-Elysee et al. (2012) [16]	+	+	+	+	?	+	?
Bergeron et al. (2009) [17]	+	?	+	+	-	+	-
Lunn et al. (2011) [20]	+	+	+	+	+	+	?
Backes et al. (2013) [21]	+	+	+	+	+	?	?
Sculco et al. (2015) [22]	+	+	+	+	+	+	?
Lunn et al. (2013) [23]	+	+	+	+	+	+	?
Kardash et al. (2007) [24]	+	?	+	+	+	+	?

1: sequence generation; 2: allocation concealment; 3: blinding of participants, 4: blinding of outcome assessor; 5: incomplete outcome data; 6: reporting bias, 7: other bias, +: low risk of bias; -: high risk of bias, ?: unclear risk of bias

### Primary outcomes

#### Postoperative nausea and vomiting

**Fig. 2:**
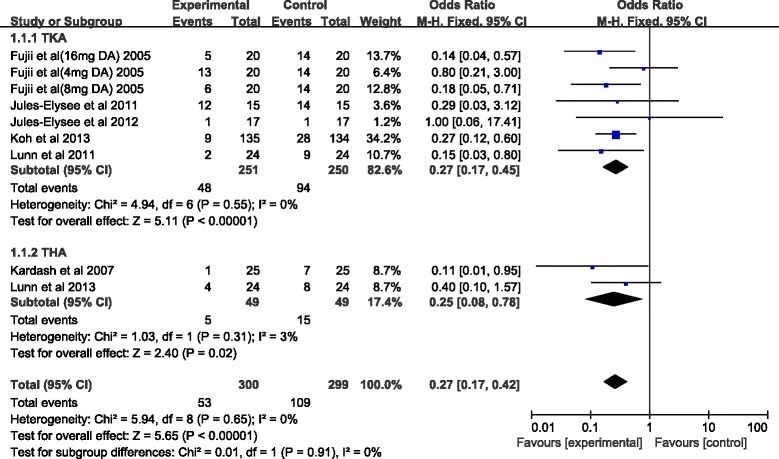
Forest plot of postoperative nausea and vomiting. Abbreviations: *95%CI* 95% confidence intervals, *df* degrees of freedom, *Fixed* fixed-effects modeling, *M-H* Mantel-Haenszel

Eight studies reported data on the occurrence of PONV [12–14, 16, 20–23]. Meta-analysis showed that systemic steroid was associated with lower risk of PONV, whether in TKA or THA (Fig. 2). This meta-analysis used a fixed-effect model because of no statistical heterogeneity among the studies (*I* ^2^ = 0%).

#### Pain

Nine studies assessed postoperative pain [12, 14, 16, 17, 20–24], though the scales and evaluation time points varied substantially. Koh et al. reported lower visual analogue scale (VAS) pain scores in the steroid group within 24 h after TKA (2.4 vs 4.0 points), but no between-group differences were found after this time point [12]. Jules-Elysee et al. showed lower VAS pain scores in the steroid group within 24 h [16] after TKA, although the differences disappeared at 48 h. Lunn and colleagues’ two studies found that methylprednisolone led to better outcomes in VAS score within 24 h after TKA and THA, but similar scores after that, until 30 days postoperatively [20, 23]. Kardash et al. showed dexamethasone caused lower numerical rating scale (NRS) on the first day after THA (1.5 vs 1.9 points) [24], and another study by Backes et al. reported superior VAS scores within 24 h after TKA and THA [21].

In contrast, another study by Jules-Elysee et al showed low-dose of systemic steroid (100 mg hydrocortisone, 2 doses each 8 h apart) to be associated with similar VAS pain scores after TKA [14], and Sculco et al. found no significant differences in average NRS scores at 24 h after THA, although the worst NRS scores were lower in dexamethasone group [22]. Bergeron and colleagues’ study showed similar results in pain scores at 6 weeks and 1 year follow-up [17].

#### Complications and adverse events

All studies reported the occurrence of potential perioperative complications and adverse events. All studies showed the complication of infection in TKA and THA: Koh et al reported two cases of periprosthetic joint infection (one in each group) [12] and Backes and colleagues’ study reported one superficial stitch abscess in steroid group in TKA [21], and other studies did not show any infection cases. All studies assessed delayed wound healing: Koh et al. reported two cases of delayed wound healing in steroid group and three cases in control group in TKA [12], and this complication was not founded in any other studies. Two studies and five studies, respectively, provided data on the potential complication of osteonecrosis of femoral head (ONFH) [17, 21] and venous thromboembolic events (VTE) [15, 20–23]: except two DVTs (one in control group and one in steroid group) reported by Backes and colleagues [21], the related studies didn’t find any ONFH or VTE cases within follow-up. In addition, five studies each assessed the adverse event of pruritus [14, 16, 20, 22, 24] and the levels of serum glucose on the operation day [14, 16, 20–22].

Meta-analysis showed that both steroid group and control group were associated with similar low risk of superficial or deep infection, delayed wound healing, ONFH, venous thromboembolic events (VTE) and pruritus. However, systemic steroid was associated with higher levels of serum glucose on the operation day, whether in TKA or THA (TKA: WMD = 22.56 mg/dl, 95% CI 16.9~28.23, *P* < 0.00001; THA: WMD = 29.45 mg/dl, 95% CI 8.06~50.84, *P* = 0.007). Table 4 shows the detailed descriptions of all the perioperative complications and adverse events.

**Table 4:** The results of meta-analyses in complications

Complications and adverse events	Surgery	Studies (*n*)	*P* value	Incidence		
				Odds ratio (95% CI)	Heterogeneity (*I* ^2^)	Model
Infection	TKA THA	6 5	0.69 -	1.51 [0.19, 11.84] -	0% -	Fixed -
Delayed wound healing	TKA THA	6 5	0.66 -	1.50 [0.25, 9.12] -	0% -	Fixed -
ONFH	TKA THA	1 2	- -	- -	- -	- -
VTE	TKA THA	4 2	- -	- -	- -	- -
Pruritus	TKA THA	3 2	0.56 0.65	1.41 [0.44, 4.49] 1.53 [0.24, 9.59]	0% 0%	Fixed Fixed
Serum glucose	TKA THA	4 2	0.001 0.007	22.56 mg/dl [16.9, 28.23]* 29.45 mg/dl [8.06, 50.84]*	0% 0%	Fixed Fixed

*ONFH* osteonecrosis of femoral head, *VTE* venous thromboembolic events

-Not occurring, unable to statistically analyze

*Were meta-analyzed in terms of the weighted mean difference (WMD)

### Secondary outcomes

#### Postoperative function

Four studies reported functional outcomes [14, 16, 17, 21], though functional scales and follow-up periods varied substantially. Two studies by Jules-Elysee et al. showed better ROM in steroid group at discharge after TKA [14, 16], and Backes and colleagues’ study found that patients given systemic dexamethasone ambulated significantly farther on postoperative day 0, 1, and 2 versus controls [21] after TKA and THA. In contrast, study by Bergeron et al. showed similar HHS scores at 6 weeks or 1 year follow-up after THA.

#### Hospitalization time

**Fig. 3:**
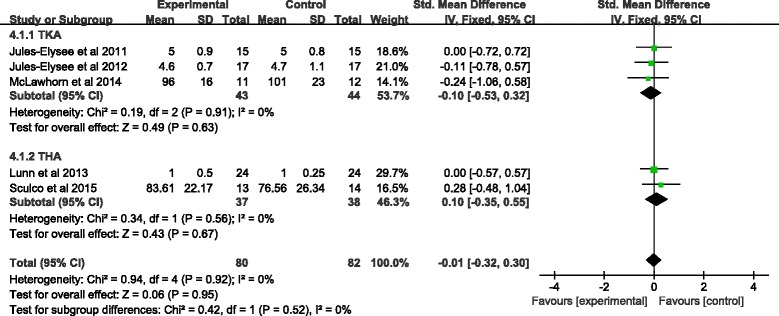
Forest plot of hospital length of stay. Abbreviations: *95%CI* 95% confidence intervals, *df* degrees of freedom, *Fixed* fixed-effects modeling, *IV* inverse variance

Six studies exactly evaluated this outcome [14–16, 20, 22, 23]. Standardized mean difference (SMD) was used because data was reported in different ways across studies. Meta-analysis showed similar results in LOS between groups, whether in TKA or THA (TKA: SMD = −0.1, 95%CI −0.53~0.32, *P* = 0.63; THA: SMD = 0.10, 95%CI −0.35~0.55, *P* = 0.95) (Fig. 3).

#### Inflammatory factors

Six studies reported data on inflammatory factors of IL-6 or CRP at varied time points [14, 16, 20, 22–24], the outcomes were synthesized qualitatively. Jules-Elysee and colleagues, respectively, found that dexamethasone led to lower levels of IL-6 within 12 h [14] and 24 h [16] after TKA. Two studies by Lunn et al. showed lower serum CRP concentrations in steroid groups at postoperative 24 h, whether in TKA [20] or THA [23]. Kardash et al. showed that systemic steroid was associated with a lower level of CRP at postoperative 48 h after THA, and another study by Sculco et al. also reported a better result in serum IL-6 concentration within 24 h after THA.

#### Thrombogenic markers

Two studies, respectively, reported data on this outcome in TKA [15] and THA [22], which in terms of plasmin-alpha-2-antiplasmin complex (PAP) and prothrombin fragme1.2 (PF1.2). McLawhorn et al found that the steroid group had significantly lower mean PAP and PF1.2 compared to controls [15]. However, unlike TKA, the serum PAP and PF1.2 concentrations were not statistically different between groups after THA [22].

## Discussion

This is the first systematic review and meta-analysis looking at perioperative systemic steroid administration for rapid recovery in patients undergoing TKA/THA. The current evidence suggests systemic steroid may be effective to reduce PONV and postoperative acute pain (within 24 h) without sacrificing the safety. In addition, the preliminary results also show the superior possibility of systemic steroid in functional rehabilitation and inflammation control, although the available evidence is still insufficient. Thus, we conclude that systemic steroid may provide faster recovery in TKA and THA, and more high-quality studies are needed to give definitive conclusion.

Several simple and straightforward interventions to manage each patient’s predictable physiologic response to surgery make patients from the sick model to the well model and achieve rapid recovery joint arthroplasty [4]. Steroid, as a kind of antiemetics and potential analgesics, is such a simple and straightforward intervention to reduce PONV and relief pain to make arthroplasty patients from sick to well.

Through the central antiemetic effect, steroid achieves the reduction of PONV by systemic application rather than topical application [25]. However, it is still unclear about the relationship between the doses of systemic steroid and PONV control. Previous studies showed that an effective dose should be dexamethasone 5 mg at least or other equivalent steroid in reducing PONV during various surgeries [26, 27], and this may be a reference to joint arthroplasty. Generally, a dose of 0.1 mg/kg dexamethasone is the distinction between high and low doses [25]. In our study, despite available data is insufficient to perform subgroup meta-analysis for dose–response relationship, studies with low-dose steroid (dexamethasone ≤ 0.1 mg/kg) reported similar unsatisfactory results between groups in anti-vomitting [14, 16]. On the other hand, all studies with high-dose steroid (dexamethasone > 0.1 mg/kg) showed lower incidence in PONV [12, 13, 15, 17, 20–24]. Thus, the current evidence preliminary suggests high-dose of systemic steroid is necessary to effectively reduce PONV in TKA/THA, and further research is needed to examine the exact dose in greater detail.

Steroid provides pain relief by reducing postoperative inflammation [7, 28]. In our meta-analysis, effective pain relief was associated with high-dose systemic steroid (dexamethasone > 0.1 mg/kg) [12, 20, 21, 23, 24], but the two studies with low-dose systemic application provided inconsistent results [14, 16]. Thus, our meta-analysis suggests that only high-dose systemic steroid was effective in reducing postoperative pain in arthroplasty, which is consistent with previous studies [25, 29]. In addition, we can find that systemic steroid provides effective pain relief only within 24 h after operation, and meanwhile, serum inflammatory factors of IL-6 and CRP are also well controlled in steroid groups during this period [14, 16, 20, 22–24], so it confirms that pain reduction with steroid is indeed associated with the inflammation control. Therefore, our meta-analysis of available clinical evidence supports that in practice, systemic steroid provides effective pain control only with 24 h after TKA/THA, and no adequate evidence supports longer pain relief with systemic steroid.

While chronic steroid increases the risks of infection, gastric ulcer and osteonecrosis in general patients, previous studies have revealed that a perioperative single dose of steroid did not increase complications in surgical patients [27, 29, 30]. A systemic review included 51 RCTs in trauma surgery showed that a single administration of high-dose methylprednisolone was not associated with a significant increase in any adverse effects [27], and another meta-analysis included 24 RCTs of a single dose systemic dexamethasone in various types of surgeries also showed the same result [29]. For arthroplasty, infection is a kind of recognized disastrous complication, the concerns regarding potential infection still prevent wide-ranging use of steroid in arthroplasty at present. In this meta-analysis, six and five studies, respectively, provided the incidence of infection in TKA and THA. Except Koh et al. and Backes et al. reported very few infection cases, other studies provided zero infection results within follow-up periods from 1 month to 1 year, and our meta-analysis also showed no difference in infection (OR = 1.51, 95% CI: 0.19~11.84). Thus, the preliminary result shows that systemic steroid may not increase the risk of infection in TKA and THA. However, due to such a low incidence, a sample size of at least 3500 patients are needed to detect a 1.5% difference in periprosthetic infection in TKA for prospective study [31]. Therefore, as the samples of included studies in our meta-analysis are quite small (range from 23 to 269), the difference of infection between groups is very difficult to be accurately evaluated in fact. In addition, another important potential risk of ONFH also makes surgeons concern a lot. In our meta-analysis, despite two studies [17, 21] reported this complication within zero-risks at 6 months and 1 year follow-up, the fairly low incidence also made the result hard to be assessed. More importantly, the follow-up periods were too short to find potential ONFH cases, which might occur several years after steroid administration. Accordingly, although our result shows that systemic steroids do not increase the risks of infection and ONFH in TKA and THA, more large-scale safety studies are warranted to give definitive conclusions

Our meta-analysis suggests a higher level of postoperative serum glucose on the operation day, whether in TKA or THA, but such difference is in fact not clinically significant unless it is particularly bothersome to severe diabetic patients.

An interesting result is that systemic steroid may suppress thrombogenic markers concentration after TKA [15]. The complex mechanism involves inflammatory reaction, coagulation cascade, and ischemical reperfusion injury (IRI), which can be simply viewed as the result of effective inflammation control with systemic steroid [32, 33]. The authors also suggested that perioperative systemic steroid may play a positive role in VTE prophylaxis following TKA.

Our meta-analysis has limitations. Firstly, as mentioned above, the sample size is too small to accurately assess the complications. This is also an important limitation in other meta-analyses of steroid injection as well [7, 27, 29]. Lacking of adequate data to perform a subgroup analysis of dose–response relationship is another important limitation, and more clinical study is warranted to recommend optimal dose.

## Conclusions

In summary, our study suggests that by providing lower incidence of PONV and less postoperative acute pain (within 24 h), high-dose systemic steroid (dexamethasone > 0.1 mg/kg) may play a critical role in rapid recovery to TKA and THA. In addition, the preliminary results also show the superior possibility of systemic steroid in functional rehabilitation and inflammation control, although the available evidence is still insufficient. However, the limitations of our meta-analysis highlight the need for large, high-quality studies that investigate the complications and dose–response relationship to give final conclusions.

## Additional file

Additional file 1: Figure S1. Detailed search histories for Pubmed. **Figure S2.** Detailed search histories for OVID. **Figure S3.** Detailed search histories for Web of Science databases. (ZIP 46 kb)

## Abbreviations

HHS: Harris hip score
IRI: Ischemial reperfusion injury
LOS: Hospital length of stay
NRS: Numerical rating scale
PAP: Plasmin-alpha-2-antiplasmin complex
PF1: 2 prothrombin fragment 1.2
PONV: Postoperative nausea and vomiting
RCT: Relevant randomized controlled trial
THA: Total hip arthroplasty
TKA: Total knee arthroplasty
VAS: Visual analogue scale pain score
