# Supplementary figures and images for: Perioperative systemic steroid for rapid recovery in total knee and hip arthroplasty: a systematic review and meta-analysis of randomized trials
Source: J Orthop Surg Res. 2017 Jun 27;12:100. doi: 10.1186/s13018-017-0601-4 (PMC5488481; doi:10.1186/s13018-017-0601-4)

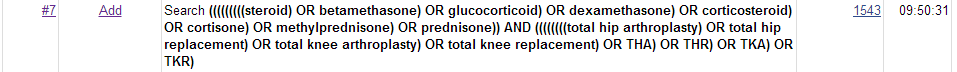

Supplement: Additional file 1: Figure S1. — Detailed search histories for Pubmed. Figure S2. Detailed search histories for OVID. Figure S3. Detailed search histories for Web of Science databases. (ZIP 46 kb) [file 13018_2017_601_MOESM1_ESM.zip › Supplementary Figure 1.png]

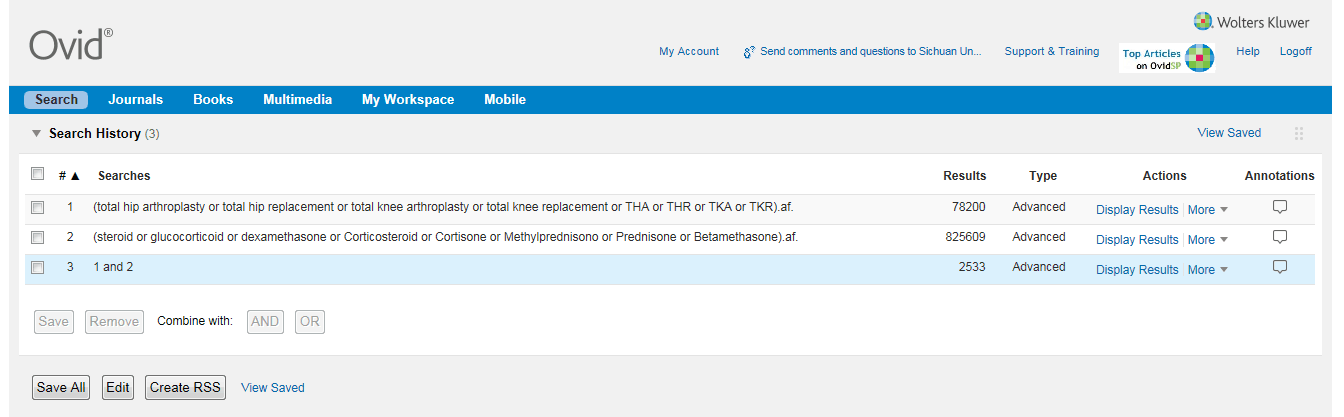

Supplement: Additional file 1: Figure S1. — Detailed search histories for Pubmed. Figure S2. Detailed search histories for OVID. Figure S3. Detailed search histories for Web of Science databases. (ZIP 46 kb) [file 13018_2017_601_MOESM1_ESM.zip › Supplementary Figure 2.png]

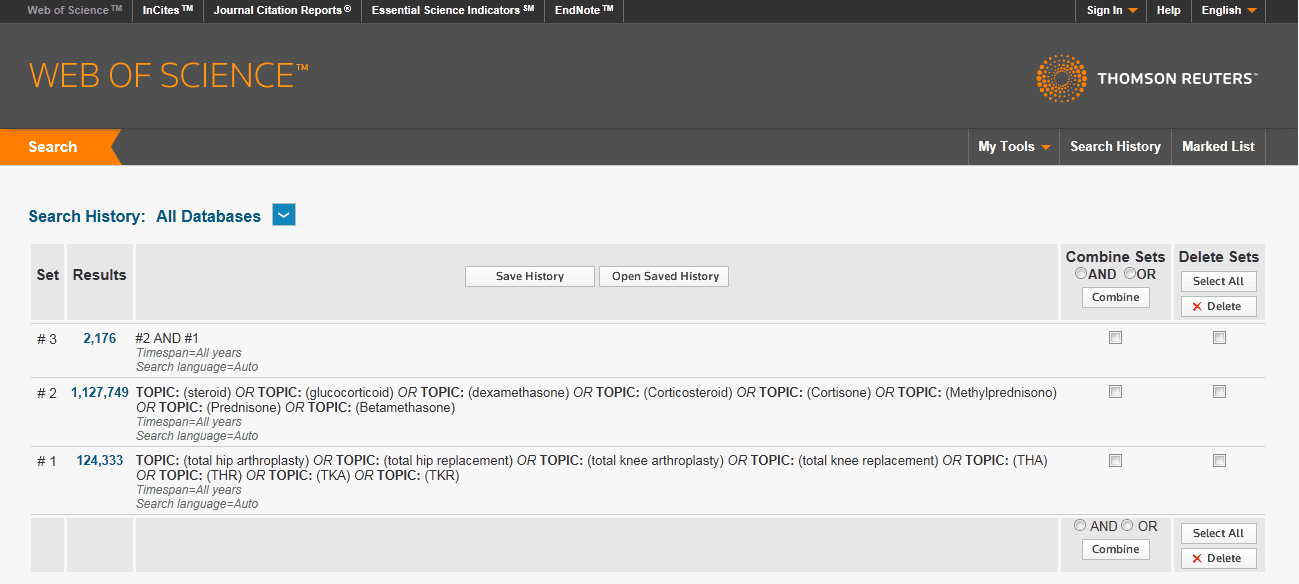

Supplement: Additional file 1: Figure S1. — Detailed search histories for Pubmed. Figure S2. Detailed search histories for OVID. Figure S3. Detailed search histories for Web of Science databases. (ZIP 46 kb) [file 13018_2017_601_MOESM1_ESM.zip › Supplementary Figure 3.png]
